# 手性金属有机骨架修饰毛细管硅胶整体柱的制备及其用于手性药物的分离

**DOI:** 10.3724/SP.J.1123.2024.01020

**Published:** 2024-11-08

**Authors:** Ruike SONG, Gan LIAO, Jiali LIN, Jialian WU, Lu HUANG

**Affiliations:** 1.福州大学化工学院, 福建 福州 350108; 1. College of Chemical Engineering, Fuzhou University, Fuzhou 350108, China; 2.闽江学院材料与化学工程学院, 福建 福州 350108; 2. College of Materials and Chemical Engineering, Minjiang University, Fuzhou 350108, China

**Keywords:** 手性金属有机骨架, 硅胶毛细管整体柱, 手性拆分, 识别机理, 分子对接, 加压毛细管电色谱, chiral metal organic frameworks (CMOF), silica monolithic capillary column, chiral separation, recognition mechanism, molecular docking, pressurized capillary electrochromatography (pCEC)

## Abstract

金属有机骨架材料(MOFs)是一类由金属离子(或金属簇)和有机配体组成的晶体结构化合物。手性MOFs作为一种新型的色谱手性分离材料,已被成功用于拆分手性对映体,表现出良好的手性拆分性能。该研究制备了一种可用于加压毛细管电色谱的手性金属有机骨架修饰毛细管硅胶整体柱。首先,合成了手性金属有机骨架(钴-甘氨酰-L-谷氨酸,Co-L-GG),接着将Co-L-GG作为手性功能单体通过一步原位聚合法制备手性毛细管整体柱。制备手性毛细管整体柱的最佳条件:Co-L-GG用量为5 mg,聚乙二醇用量为0.96 mg,四甲氧基硅烷用量为3.6 mL,甲基三甲氧基硅烷用量为0.4 mL。其次,研究了分离条件对手性药物拆分的影响。在外加电压为-20 kV、流动相为乙腈-20 mmol/L磷酸氢二钠(80∶20, v/v)的条件下,3 min内拆分了6种手性药物,其中氨氯地平、氟伐他汀和色氨酸达到了基线分离。所制备的手性毛细管整体柱表现出良好的重复性和稳定性。最后,使用AutoDock进行分子对接,并借助Discovery Studio对分子对接结果进行分析。结果显示,Co-L-GG与手性药物对映体间的结合自由能差越大,对映体选择性因子越大,然而,这并不一定导致分离度的增加。Co-L-GG中富含伯胺、仲胺和羰基,使其具有对映体识别能力。该研究表明,手性金属有机骨架可用作手性功能单体制备手性毛细管整体柱,在手性化合物的分离和分析中具有广阔的应用前景。

## Abstract (en)

Metal organic frameworks (MOFs) are crystalline compounds composed of metal ions (or metal clusters) and organic ligands. Chiral MOFs have been successfully utilized as novel materials for the separation of chiral enantiomers by chromatography, demonstrating excellent chiral separation performance. In this study, a chiral MOF-modified silica monolithic capillary column was used for pressurized capillary electrochromatography. First, a chiral MOF (Co-glycyl-L-glutamic acid, Co-L-GG) was synthesized. This MOF was then used to prepare a chiral capillary monolithic column via a one-step in situ polymerization method. The optimal conditions for preparing the chiral capillary monolithic column were determined as follows: Co-L-GG amount, 5 mg; polyethylene glycol amount, 0.96 mg; tetramethoxysilane dosage, 3.6 mL; trimethoxymethylsilane dosage, 0.4 mL. Next, the effects of the separation conditions on the separation of chiral drugs were investigated. Under the conditions of an applied voltage of -20 kV and a mobile phase consisting of acetonitrile and 20 mmol/L disodium hydrogen phosphate (80∶20, v/v), six chiral drugs were separated within 3 min, with baseline separation achieved for amlodipine, fluvastatin, and tryptophan. Moreover, the prepared chiral capillary monolithic column exhibited good reproducibility and stability. Finally, molecular docking studies were conducted using AutoDock to explore the chiral recognition mechanism, and the results were analyzed using Discovery Studio. The results indicated that larger differences in binding free energy between Co-L-GG and the enantiomers of the chiral drugs were correlated with higher enantioselectivity factors. However, this correlation did not necessarily lead to an increase in resolution. Co-L-GG, which is enriched with primary amines, secondary amines, and carbonyl groups, demonstrated enantiomeric recognition capability. In conclusion, this study demonstrates that chiral MOFs can be effectively used as chiral functional monomers to prepare chiral monolithic capillary columns, highlighting their significant potential for the separation and analysis of chiral compounds. The comprehensive exploration of the synthesis, characterization, and applications of these MOFs will help provide valuable insights into the development of advanced separation technologies.

随着手性药物的蓬勃发展,手性研究已经成为化学、材料、生物等学科的研究热点。金属有机骨架材料(MOFs)是一类由金属离子(或金属簇)和有机配体组成的晶体结构化合物。它们的结构特点是金属离子通过有机配体形成网络状的结构,形成大量的孔隙和通道。目前,MOFs已被广泛用于催化应用^[1,2]^、药物输送^[3,4]^、传感器^[5,6]^、分离富集^[7,8]^。手性MOFs作为一种新型的手性分离材料,已被成功用于液相色谱^[9,10]^、气相色谱^[11]^、毛细管电色谱^[12,13]^拆分手性对映体,且表现出良好的手性拆分性能。

加压毛细管电色谱(pCEC)是一种结合了毛细管电泳和高效液相色谱特点的混合分离技术。该方法效率高,已广泛用于复杂混合物的分离分析。手性毛细管色谱柱是pCEC手性分离分析的核心。根据固定相的制备方法,手性毛细管色谱柱可以分为3种类型:开管柱、填充柱和整体柱。与其他手性毛细管色谱柱相比,手性毛细管整体柱有很多优点,如柱效高、柱容量大,而且无需烧结柱塞。研究人员通常采用一步原位聚合法制备手性毛细管整体柱。常用的手性功能单体包括各种*β*-环糊精衍生物^[14⇓-16]^、纤维素^[17]^、胃蛋白酶^[18,19]^、手性共价有机骨架^[20]^、分子印迹聚合物^[21]^等。

目前,非手性MOFs已被证明可以辅助手性毛细管整体柱拆分手性对映体^[18]^,然而手性MOFs在手性毛细管整体柱的应用尚未见报道。另外,尽管已有许多研究涉及手性分离,但对手性固定相的对映体识别机理仍然缺乏清晰地描述^[20]^。本文将手性金属有机骨架用作手性功能单体制备手性金属有机骨架修饰毛细管硅胶整体柱(CMOF-SMCC)。首先,合成金属有机骨架钴-甘氨酰-L-谷氨酸(Co-L-GG),通过傅里叶变换红外光谱(FTIR)和粉末X射线衍射(PXRD)对Co-L-GG进行表征。然后,将Co-L-GG直接引入聚合过程,通过扫描电子显微镜(SEM)对制备的CMOF-SMCC进行表征。为实现最佳分离效果,探讨了手性毛细管整体柱的制备条件和pCEC分离条件对CMOF-SMCC手性拆分能力的影响。并将制备得到的CMOF-SMCC应用于多种手性药物的对映体分离。最后,采用AutoDock进行分子对接,并利用Discovery Studio对分子对接结果进行分析,旨在阐明CMOF-SMCC的手性识别机理。

## 1 实验部分

### 1.1 仪器与试剂

采用FTIR(8400s,日本岛津)和PXRD(SmartLab,日本理学)对Co-L-GG进行结构表征。采用SEM(SU-8010,日本日立)对毛细管整体柱进行表征。所有pCEC实验均在TriSep^®^ 3000 eHPLC (Unimicro, USA)上进行。

四甲氧基硅烷(TMOS, 98%)、甲基三甲氧基硅烷(MTMS, 98%)、甲醇(≥99.9%)、甘氨酰-L-谷氨酸(L-GG, 98%)、四水醋酸钴(99.5%)、聚乙二醇(PEG, *M*_r_ 20000)、尿素(99%),其他试剂和药品均为分析纯,以上均购于上海阿拉丁有限公司;外消旋药物标准品氨氯地平(≥99.9%)、班布特罗(99.7%)、苯丙氨酸(≥99.9%)、氟伐他汀(97.3%)、氯苯那敏(99.7%)、色氨酸(≥99.9%)购于中国食品药品检定研究院;实验用水为超纯水。石英毛细管(75 μm I.D.×365 μm O.D.)购自河北永年锐沣色谱器件有限公司。

### 1.2 Co-L-GG的合成

如图1所示,在文献[22]的基础上合成Co-L-GG。在50 mL聚四氟乙烯反应釜中依次加入150 mg四水醋酸钴、120 mg L-GG、12 mL水和12 mL甲醇,搅拌溶解并超声20 min后,将该反应釜密封置于80 ℃烘箱中反应2 h。自然冷却至室温,得到粉紫色针状晶体Co-L-GG,最后用无水乙醇洗涤5次,真空干燥后保存备用。

**图1:**
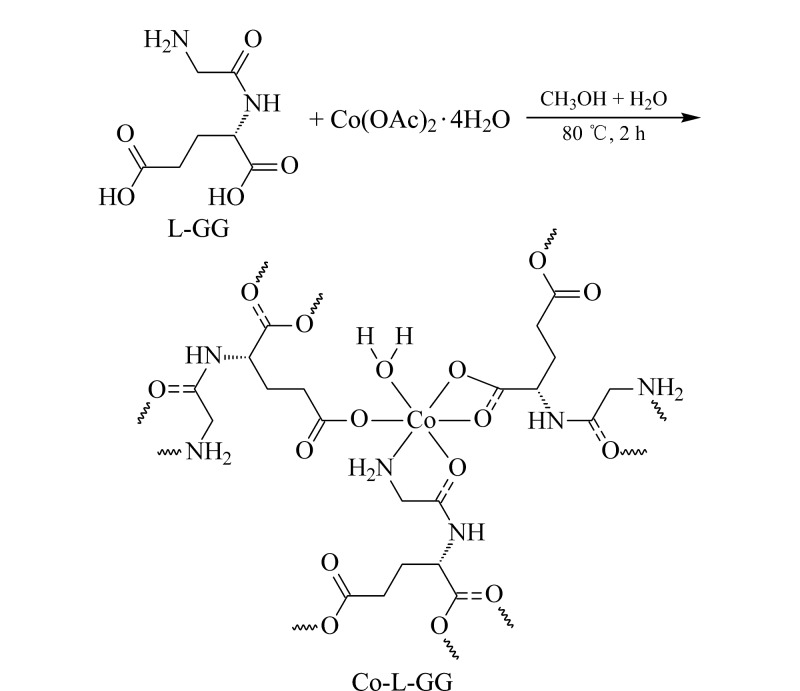
Co-L-GG的合成示意图

### 1.3 CMOF-SMCC的制备

石英毛细管依次用1 mol/L盐酸、去离子水、1 mol/L氢氧化钠溶液、去离子水、甲醇冲洗30、10、30、10、10 min,然后氮气吹干备用。将0.96 g PEG、0.90 g尿素和10 mL 0.01 mol/L乙酸水溶液在冰浴中搅拌至溶解后滴加3.6 mL TMOS和0.4 mL MTMS,搅拌45 min后得到硅溶胶。取1 mL硅溶胶加入5 mg Co-L-GG,继续在冰浴中搅拌5 min,得到紫色硅溶胶。然后将紫色硅溶胶注入毛细管至一定长度,两端用橡皮塞封口,置于40 ℃超恒温水浴锅中反应20 h。取出后去掉两端橡皮塞,放入250 ℃气相色谱仪进行25 h热处理,最后用甲醇冲洗30 min。

### 1.4 pCEC的操作条件

使用水或乙醇配制1 mg/mL手性药物母液,置于4 ℃冰箱保存,稀释至特定浓度后进样。所有溶液采用0.22 μm尼龙膜进行过滤,并通过超声波脱气。CMOF-SMCC的有效长度为25 cm,总长度为50 cm。流动相为乙腈-20 mmol/L磷酸二氢钠(80∶20, v/v)。外加电压为-20 kV,流速为0.05 mL/min,检测波长为214 nm,进样量为5 μL,反压阀1.72 MPa。

### 1.5 分子对接

由于手性识别位点只存在于Co-L-GG结构上,我们将整体柱材料简化为Co-L-GG进行分子对接。利用ChemOffice软件构建Co-L-GG和手性药物的三维分子模型。Co-L-GG作为受体分子,手性药物作为配体分子。用Avogadro软件优化初始结构。随后利用AutoDock探究受体与配体分子之间的相互作用。基于受体和配体分子中所有原子类型的相互作用势,采用Lamarckian遗传算法结合基于网格的能量评估方法计算出晶格盒(1.99 nm×5.45 nm×1.20 nm,晶格间距0.0375 nm)。总共进行了100次独立运行。在分子对接完成后,选择结合自由能最显著的配合物作为进一步分析的最优构型。

## 2 结果与讨论

### 2.1 Co-L-GG和CMOF-SMCC的表征

首先,利用FTIR对Co-L-GG和CMOF-SMCC进行表征,结果见图2a。在Co-L-GG的红外光谱图中,1655 cm^-1^处的吸收峰由仲酰胺C=O的伸缩振动产生;1577 cm^-1^处的吸收峰由仲酰胺-NH的弯曲振动产生。在含有Co-L-GG和不含Co-L-GG的整体聚合物的红外光谱图中,在1500~1700 cm^-1^范围内观察到的新波段应归因于仲酰胺C=O的伸缩振动峰和仲酰胺-NH的弯曲振动峰,表明Co-L-GG已成功修饰到硅胶整体柱上。理论上,由于Co-L-GG有羰基官能团,羰基能与硅醇发生醇酯化反应,生成硅醚。因此,Co-L-GG是通过化学修饰而不是物理修饰的方式进入硅胶毛细管整体柱。

**图2:**
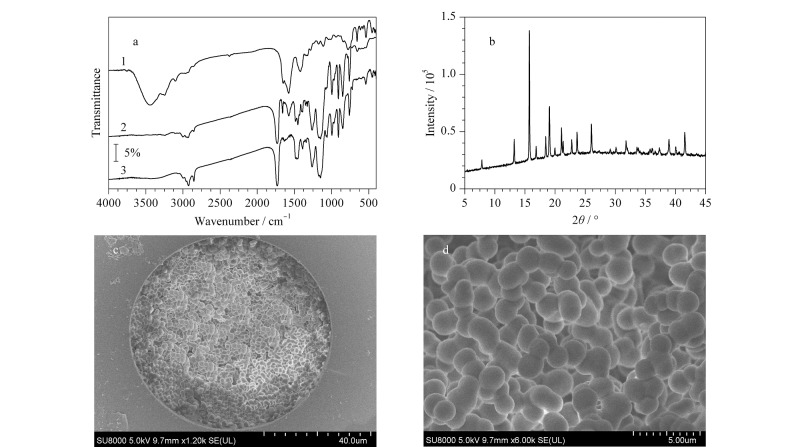
Co-L-GG和CMOF-SMCC的表征

其次,利用PXRD对Co-L-GG进行测试。如图2b所示,合成的Co-L-GG的PXRD谱图在5°~45°范围内有13.2、15.7、16.8、18.4、19.0、20.0、21.1、21.4、22.7、23.6、26.0、31.8、38.9、41.5等特征峰,与文献[22]报道的数据基本一致。上述FTIR和PXRD结果均表明Co-L-GG已成功合成。最后,通过SEM对CMOF-SMCC的横截面进行表征。如图2c和图2d所示,可以观察到完整均匀的整体柱基体,微球连续,孔隙致密,且整体基质很好地附着在毛细管内壁上。上述结果说明Co-L-GG已修饰到硅胶整体柱中,CMOF-SMCC制备成功。

### 2.2 制柱条件的优化

为了找到制备CMOF-SMCC的最佳条件,以氨氯地平为拆分对象,对Co-L-GG的用量、PEG的用量以及TMOS与MTMS的比例进行了优化。

首先,制备了Co-L-GG用量分别为2、5和8 mg的CMOF-SMCC。实验结果表明,随着Co-L-GG用量的增大,柱压逐渐增大。当Co-L-GG用量为2 mg和8 mg时,CMOF-SMCC没有手性拆分效果;Co-L-GG用量为5 mg时,氨氯地平得到了较好的拆分(见附图S1,www.chrom-China.com)。

其次,考察了PEG用量分别为0.80、0.88、0.96和1.00 g时CMOF-SMCC的拆分效果。当PEG用量为0.80、0.88和1.00 g时,CMOF-SMCC没有手性拆分效果;PEG用量为0.96 g时,氨氯地平得到了较好拆分(见附图S2)。

最后,在TMOS与MTMS总用量为4 mL的基础上,探讨了TMOS与MTMS的体积比对CMOF-SMCC手性拆分效果的影响。当不加入MTMS时,CMOF-SMCC没有手性拆分效果;而TMOS与MTMS的体积比为3∶1、5∶1和9∶1时,随着体积比的增大,氨氯地平的分离度越来越大(见附图S3)。

综上所述,制备CMOF-SMCC的最佳条件如下:Co-L-GG用量为5 mg, PEG用量为0.96 mg, TMOS用量为3.6 mL, MTMS用量为0.4 mL。

### 2.3 手性药物的拆分

为了找到拆分手性药物的最佳条件,以氨氯地平为拆分对象,对进样端外加电压、流动相组成进行了优化。

首先,考察了外加电压分别为-5、-10、-15和-20 kV时CMOF-SMCC的拆分效果。随着外加电压绝对值的增大,氨氯地平的分离度越来越大(见附图S4)。这是因为所加电压为负电压,电渗流方向与流动相运行方向相反,随着电压绝对值的增加,电渗流增大,氨氯地平对映体受到电渗流的作用力也变大,因而降低了氨氯地平对映体的表观淌度,使出峰时间稍有延长,分离度得到提高。由于-20 kV是仪器所能施加的最小负电压,因此选择-20 kV作为外加电压。

其次,考察了不同体积比的乙腈与磷酸二氢钠溶液(20 mmol/L)作为流动相时CMOF-SMCC的拆分效果。当乙腈体积分数低于60%时,样品出峰时间过快导致对映体无法很好拆分;当乙腈体积分数为100%时,CMOF-SMCC没有任何手性拆分效果;而当乙腈体积分数为80%时,对映体获得了很好的拆分(见附图S5)。

综上,拆分氨氯地平的最佳条件如下:外加电压为-20 kV,流动相为乙腈和20 mmol/L磷酸氢二钠(80∶20, v/v)。

在上述拆分条件下,选择不同的手性药物(班布特罗、苯丙氨酸、氟伐他汀、氯苯那敏、色氨酸)作为目标分析物考察了CMOF-SMCC的手性选择性。实验结果如图3所示,在3 min内,氨氯地平得到了基线拆分,氟伐他汀、色氨酸、班布特罗、氯苯那敏和苯丙氨酸得到了部分拆分。上述结果证实了CMOF-SMCC的手性分离能力。

**图3:**
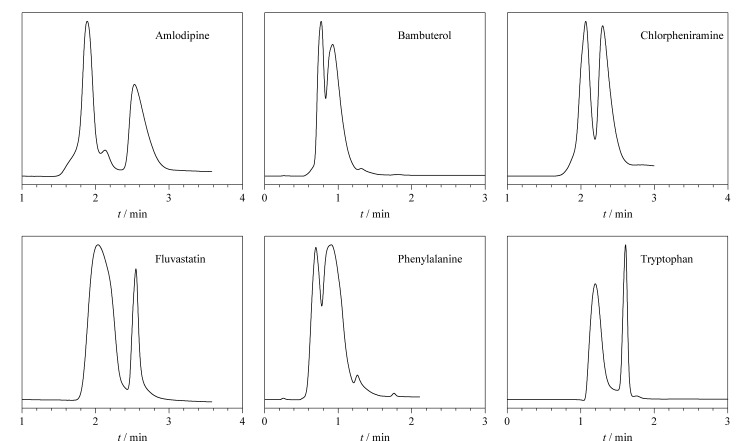
手性药物(0.1 mg/mL)的手性拆分

以氨氯地平为被测物,以保留时间和分离度的RSD值评价CMOF-SMCC的重复性和稳定性(见附表S1)。可以看出,日内、日间、柱间的RSD值分别为2.6%~4.1%、2.8%~4.6%、3.4%~5.8%。此外,CMOF-SMCC在进样100针以后仍然维持良好的分离度和峰形。上述结果表明CMOF-SMCC具有良好的稳定性和重复性。

### 2.4 手性识别机理

为了揭示CMOF-SMCC对手性药物的识别机理,采用分子对接研究了Co-L-GG与分离度较好的4种手性药物之间的相互作用。表1给出了结合自由能(Δ*G*)、结合自由能差(|ΔΔ*G*|)、对映体选择性因子(*α*)、分离度(*R*_s_)和分子间相互作用(不包括范德华力)。主体-客体复合物的构型在图4(使用Discovery Studio创建)和附图S6(使用Avogadro创建)中显示。从表1可以看出,Co-L-GG与手性药物对映体之间的结合自由能差越大,手性药物的对映体选择性因子也越大,而分离度却并不一定越大。客体和主体之间的结合自由能值的变化主要来自不同分子间相互作用的数量和类型,如氢键、疏水相互作用、*π*-*π*相互作用和离子-*π*相互作用等。分子间相互作用强度和主体-客体复合物的稳定性均随着结合自由能绝对值的增加而增加。与*S*-氨氯地平相比,Co-L-GG与*R*-氨氯地平之间多了一个氢键,少了一个*π-σ*相互作用。通常,氢键比*π-σ*相互作用更强,因此Co-L-GG与*R*-氨氯地平之间的相互作用更大。与*S*-氯苯那敏相比,Co-L-GG与*R*-氯苯那敏之间多了2个氢键,因此相互作用更强。与*R*-氟伐他汀相比,Co-L-GG与*S*-氟伐他汀多了2个氢键,少了一个*π-σ*相互作用,因此相互作用也更强。与D-色氨酸相比,Co-L-GG与L-色氨酸之间多了3个氢键,少了1个*π-σ*相互作用,因此相互作用更强。此外,图4还说明了Co-L-GG的伯胺、仲胺和羰基在主体-客体相互作用中发挥着关键作用,对其对映体分离能力有显著贡献。

**表1:** Co-L-GG与对映体的结合自由能、结合自由能差、*α*、*R*_s_和分子间相互作用(范德华力除外)

Analyte	Δ*G*/(kcal/mol)	|ΔΔ*G*|/(kcal/mol)	*α*	*R*_s_	Molecular interactions
*R*-Amlodipine	-3.28	0.35	1.39	1.72	three hydrogen bondings
*S*-Amlodipine	-2.93				two hydrogen bondings, and one *π-σ* interaction
*R*-Chlorpheniramine	-5.93	0.19	1.17	0.69	six hydrogen bondings
*S*-Chlorpheniramine	-5.74				four hydrogen bondings
*R*-Fluvastatin	-4.58	0.27	1.28	1.11	three hydrogen bondings, and one *π-σ* interaction
*S*-Fluvastatin	-4.85				five hydrogen bondings
D-Tryptophan	-3.52	0.37	1.41	1.21	two hydrogen bondings, and one *π-σ* interaction
L-Tryptophan	-3.89				five hydrogen bondings

**图4:**
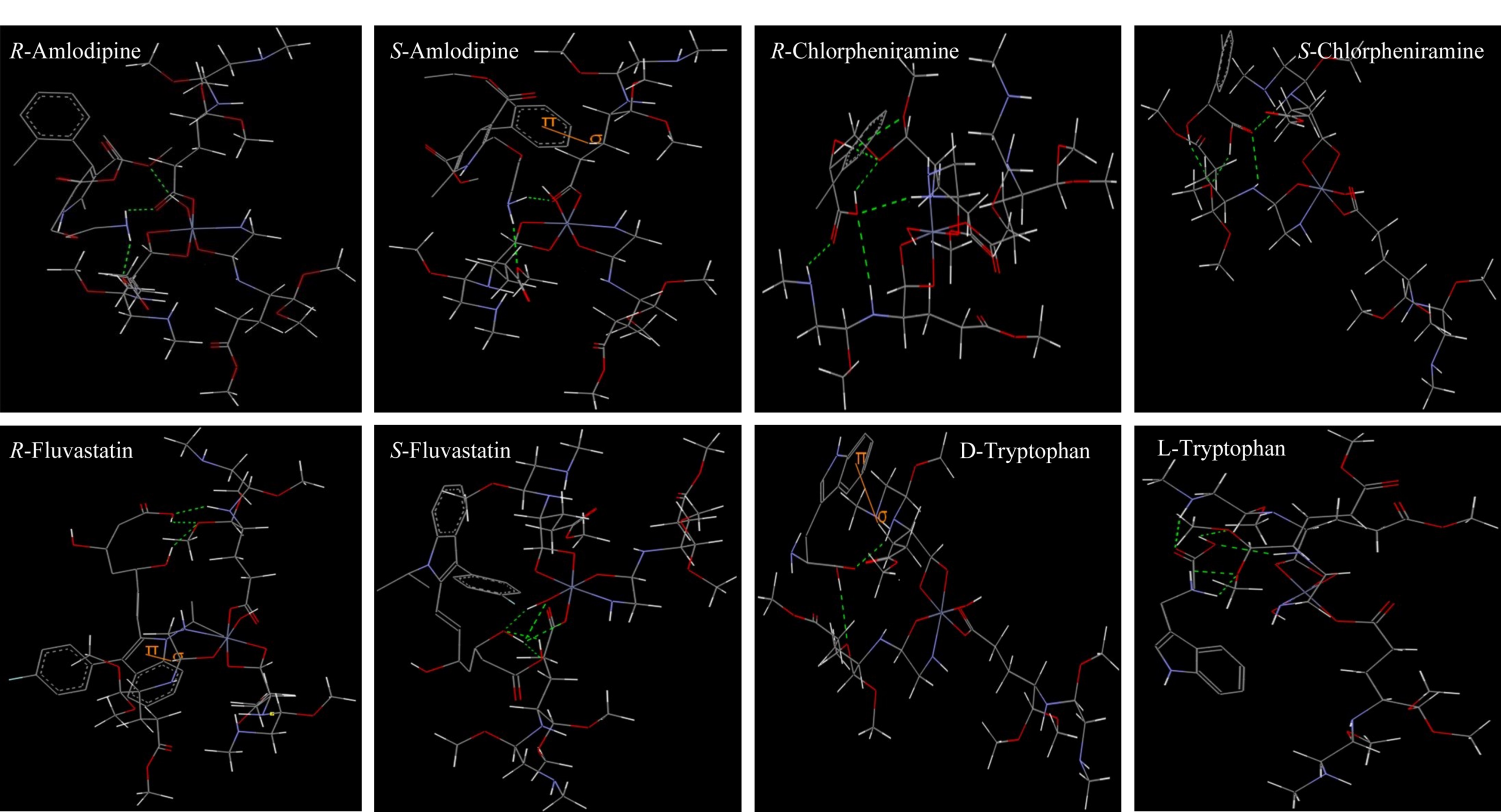
通过Discovery Studio分析主体-客体复合物构型

## 3 结论

该研究成功制备了一种新型的手性金属有机骨架修饰毛细管硅胶整体柱,并对其进行了表征。通过对手性毛细管柱的制备条件和分离条件的优化,在3 min内完全拆分了氨氯地平,基线拆分了氟伐他汀和色氨酸,并部分拆分了班布特罗、氯苯那敏和苯丙氨酸。所制备的手性毛细管整体柱具有良好的重复性和稳定性。此外,利用分子对接以Co-L-GG为受体研究了手性毛细管整体柱的识别机理。研究结果表明,Co-L-GG与手性药物的结合自由能差越大,对映体选择性因子越大,尽管分离度并非一定随之增加。Co-L-GG中富含伯胺、仲胺和羰基,赋予其出色的对映体识别能力。这项研究揭示了手性金属有机骨架可作为制备手性毛细管整体柱的手性功能单体,为手性化合物的分离和分析提供了广泛的应用前景。
